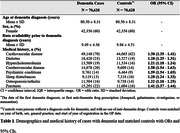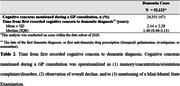# Cognitive symptoms and medical history prior to mild cognitive impairment and dementia diagnosis: a retrospective case‐control study using Dutch primary care data

**DOI:** 10.1002/alz.090705

**Published:** 2025-01-09

**Authors:** Brenda N. Baak, Casper de Boer, Mahsa Nooralishahi, Argonde C. van Harten, Everard G.B. Vijverberg, Wiesje M. van der Flier, Ron M.C. Herings

**Affiliations:** ^1^ PHARMO Institute for Drug Outcomes Research, Utrecht Netherlands; ^2^ Alzheimer Center Amsterdam, Neurology, Vrije Universiteit Amsterdam, Amsterdam UMC location VUmc, Amsterdam Netherlands; ^3^ Amsterdam Neuroscience, Neurodegeneration, Amsterdam Netherlands; ^4^ Medisch Centrum Mediport, Amsterdam Netherlands; ^5^ Amsterdam Public Health, Methodology, Amsterdam Netherlands; ^6^ Department of Epidemiology and Data Science, Amsterdam UMC, Amsterdam Netherlands

## Abstract

**Background:**

General practitioners (GPs) play a crucial role in recognizing cognitive deficits and diagnosing dementia. Currently, dementia diagnosis in primary care is prone to be missed or delayed. Electronic health records from GPs can offer insights into the trajectory leading up to a dementia diagnosis. Our aim was to characterize prior medical history of health conditions known to be associated with dementia and cognitive concerns in dementia cases and controls using Dutch primary care data.

**Method:**

In this retrospective case‐control study using data from the ABOARD‐GP Cohort (2011‐2022), we identified dementia cases and an equal number of controls (matched on birth year, sex, general practice and registration year). A prior medical history of cardiovascular disease, diabetes, hypercholesterolemia, cerebrovascular disease, psychiatric conditions, sleep disturbances, osteoporosis/arthritis, and fracture was assessed. Cognitive concerns mentioned during a GP consultation was operationalized as mentioning of (1) memory/concentration/orientation complaints/disorders, (2) overall decline, and/or (3) Mini‐Mental State Examination. Logistic regression compared each condition in medical history between dementia cases and controls. Time from first recorded cognitive concern to dementia diagnosis was estimated.

**Result:**

We identified 70,628 dementia cases and 70,628 controls (mean age 80 years, 60% female) (Table 1). We found significant associations between all conditions in medical history and a dementia diagnosis. Cerebrovascular disease and psychiatric conditions, the two strongest associations, corresponded to a 58% and 59% higher odds of having a dementia diagnosis. Prior cognitive concerns mentioned during GP consultations were recorded in 47% of dementia cases (Table 2), indicating that over half had no documented history of cognitive concerns. For those with mentioning of a cognitive concern, the mean time from first cognitive concern to diagnosis was 2.14 years.

**Conclusion:**

This study using GP data shows that cases with dementia were more likely to have pre‐existing health conditions than controls. One hypothesis for this could be that these conditions are known risk factors for dementia. Alternatively, individuals with these other conditions have previously sought medical attention from a GP, facilitating the detection of cognitive concerns. These results underscore the importance of timely recognition of cognitive symptoms and addressing risk factors to reduce the impact on cognitive health.